# National Association of Dental Examiners

**Published:** 1890-09

**Authors:** 


### NATIONAL ASSOCIATION OF DENTAL EXAMINERS.

THIS important organization met at Excelsior Springs, Mo., August 4.

A large portion of the proceedings of this body are, necessarily, of a character unsuited for publication, as they involve frequently the interests of individuals and institutions.

This organization derives its power to act from the State Examiners, and has not intrinsically any legal status. Its power is, however, none the less on this account, as its orders go down to the State Boards, and through them they acquire legal force. This necessitates the greatest possible care in the consideration and administration of its work.

It is to be regretted that this was not manifested at the recent session. The attempt to arraign certain colleges for a supposed dereliction of duty was certainly outside of the general work of this organization. This would properly seem to be the province of the legally organized body, to whom all complaints should be relegated for examination.

The National Association, however, thought otherwise, and assumed to try these colleges in secret session and without notice to the supposed offenders. The charge originated from a single absent member of the Association, and was simply a general statement without a particle of evidence to substantiate it. Had this been presented, the charge was of such a puerile nature that no body of dignified character would for a moment have entertained it, certainly not without giving due notice to the parties implicated.

The present condition of affairs between the colleges and State Examiners is not altogether satisfactory, and it is imperative on both sides to move with caution. There is no necessity for antagonism; but if there is to be a repetition of this kind of crude legislation it will certainly fan the smothered embers into a flame of righteous indignation that can only end in injury to dental education in this country.

